# *Brucella* BtpB Manipulates Apoptosis and Autophagic Flux in RAW264.7 Cells

**DOI:** 10.3390/ijms232214439

**Published:** 2022-11-20

**Authors:** Junmei Li, Lin Qi, Ziyang Diao, Mengyu Zhang, Bin Li, Yunyi Zhai, Mingyue Hao, Dong Zhou, Wei Liu, Yaping Jin, Aihua Wang

**Affiliations:** 1 College of Veterinary Medicine, Northwest A&F University, Yangling District, Xianyang 712100, China; 2 Key Laboratory of Animal Biotechnology of the Ministry of Agriculture, Northwest A&F University, Yangling District, Xianyang 712100, China

**Keywords:** *B. suis* S2, BtpB, apoptosis, autophagy, autophagic flux, intracellular survival

## Abstract

*Brucella* transfers effectors into host cells, manipulating cellular processes to its advantage; however, the mechanism by which effectors regulate cellular processes during infection is poorly understood. A growing number of studies have shown that apoptosis and autophagy are critical mechanisms for target cells to cope with pathogens and maintain cellular homeostasis. BtpB is a *Brucella* type IV secretion system effector with a complex mechanism for manipulating host infection. Here, we show that the ectopic expression of BtpB promoted DNA fragmentation. In contrast, an isogenic mutant strain, Δ*btpB*, inhibited apoptosis compared to the wild-type strain *B. suis* S2 in RAW264.7 cells. In addition, BtpB inhibited autophagy, as determined by LC3-II protein levels, the number of LC3 puncta, and p62 degradation. We also found that BtpB reduced autophagolysosome formation and blocked the complete autophagic flux. Moreover, our results revealed that the autophagy inhibitor, chloroquine, reduces *Brucella*’s intracellular survival. Overall, our data unveil new mechanisms of virulence implicating the effector BtpB in regulating host intracellular infection.

## 1. Introduction

Brucellosis is a severe global zoonosis causing abortion and sterility in ruminant animals and debility in humans [1,2]. Humans contract brucellosis primarily through ingestion of *Brucella*-contaminated milk and dairy products or direct contact with infected animals [3]. Brucellosis causes great economic losses, especially in the breeding industry, and public health concerns [4]. Therefore, it is urgent to clarify the pathogenic mechanisms of *Brucella*, which may help to prevent and control brucellosis effectively.

*Brucella* has evolved several stealthy strategies to survive and establish a replicative niche in macrophages and dendritic cells, which is the basis for reaching tissues and establishing systemic infection [5]. After internalization on lipid rafts, the microbe resides in *Brucella*-containing vacuoles (BCV) and where it undergoes transient fusion with lysosomes and acidification [6,7]. This process is necessary to activate the type IV secretion system (T4SS). T4SS translocates a variety of effector proteins into host cells and interferes with specific host functions to enable the bacteria to survive, persist, and multiply [8,9,10]. BtpB is a *Brucella* T4SS effector protein containing a TIR domain that serves as a scaffold for TLRs and related adaptor molecules. Several studies have proven that BtpB can escape the host immune response by inhibiting the TLR pathway [11,12,13]. However, the other molecular functions and targets of BtpB are still worth exploring.

As a conserved degradation process, autophagy maintains cellular homeostasis and plays a critical role in various diseases, including infection by pathogens [14]. Autophagy is an intracellular clearance process. First, the substrates are wrapped in a double membrane structure to form an autophagosome and then transported to the lysosome forming a degradative autophagolysosome [15]. After degradation of the autophagic contents, the products in autophagolysosomes are released, and lysosomes are reformed [16]. This complete dynamic process is termed autophagy flux [17]. While autophagy limits exogenous infection, bacterial pathogens have evolved mechanisms to interfere with autophagic initiation or autophagy flux for their own benefit [18,19]. For example, *Salmonella* effector AvrA inhibits Beclin-1 protein expression through the JNK pathway and reduces the autophagic response to benefit *Salmonella* survival [20]. The *C. burnetii* protein, CvpF, manipulates endosomal trafficking and autophagy induction for optimal vacuole biogenesis [21]. The *B. abortus* A19 T4SS virB promoter mutant strain, ΔvirB, showed increased autophagy, upregulated IL-6, and reduced survival in macrophages and dendritic cells [22]. Another study showed that deletion of the T4SS effector, VceA, of *B. abortus* could promote autophagy and upregulate IL-1β and TNF-α in human trophoblast cells during *Brucella* infection [23]. In addition, several virulence factors (such as Omp31 and LysR) from *Brucella* have been shown to alter autophagy in macrophages, but the molecular mechanism remained largely unexplored [24,25]. Thus, determining the regulatory roles and molecular mechanisms of *Brucella* virulence factors in the host autophagy process is crucial for providing a new view of the pathogenic mechanism.

In this study, we used multiple methods to demonstrate that the *Brucella* T4SS effector, BtpB, could promote apoptosis and found that the mutant strain, Δ*btpB*, activated autophagy, promoted autophagolysosome formation, and facilitated autophagy flux in the RAW264.7 macrophage cell line. Furthermore, Δ*btpB* restrained intracellular pathogen replication when the pH of the lysosome was increased with chloroquine. Our findings provide new insights on BtpB manipulation of host functions.

## 2. Results

### 2.1. Brucella BtpB Induces Apoptosis in RAW264.7

**Figure 1:**
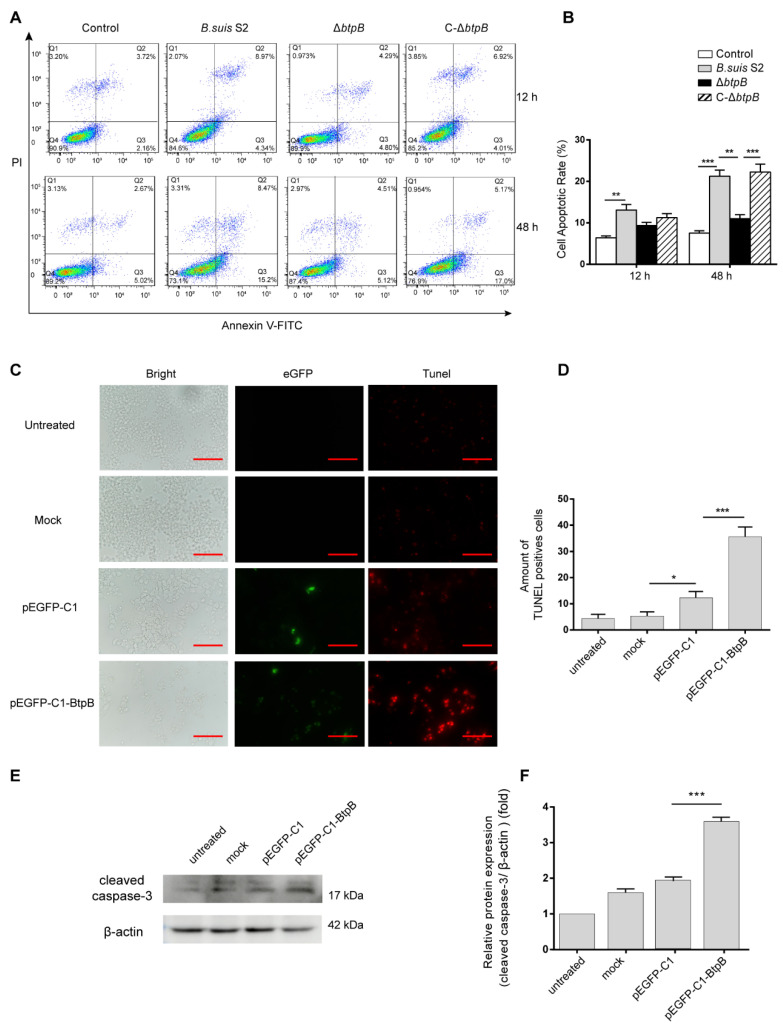
BtpB induces apoptosis in RAW264.7. (**A**) RAW264.7 cells were infected with *B. suis* S2, Δ*btpB*, or C-Δ*btpB* (MOI = 200) for 12 and 48 h. The cells were stained with Annexin V-FITC and PI and then detected by flow cytometry. (**B**) Percentage of apoptotic cells. (**C**) RAW264.7 cells were transfected with pEGFP-C1 or pEGFP-C1-BtpB for 48 h and were labeled with TUNEL. Scale bars, 100 μm. (**D**) Number of TUNEL-positive cells at the indicated times. (**E**) RAW264.7 cells were transfected with pEGFP-C1 or pEGFP-C1-BtpB for 48 h and the cleaved caspase-3 and β-actin were detected by immunoblotting analysis. (**F**) The ratio of cleaved caspase-3 relative to β-actin levels was calculated using ImageJ. The experiment was repeated three times and data represent mean ± SD. Results were analyzed with one-way ANOVA and post-hoc Bonferroni multiple comparison test. * *p* < 0.05; ** *p* < 0.01; *** *p* < 0.001.

Modulation of apoptosis is a common survival strategy used by infectious agents. *Brucella* T4SS and its effectors play a major part in regulating the apoptosis of infected cells [23,26]. To determine whether BtpB affects apoptosis during *Brucella* infection, we measured the apoptosis rate in RAW264.7 cells infected with *B. suis* S2, Δ*btpB*, or C-Δ*btpB* and labeled with Annexin V and PI using a flow cytometer. The results indicated that the average number of early apoptotic cells (Annexin-V-positive) from the Δ*btpB*-infected group decreased significantly at 48 h post-infection, reaching 5.12%. The average apoptotic rates of early apoptotic cells with *B. suis* S2 and C-Δ*btpB* were 15.2% and 17.0%, respectively (Figure 1A,B). The results show that Δ*btpB* inhibits apoptosis of RAW264.7 at 48 hpi. To further verify the pro-apoptotic effect of BtpB, we constructed pEGFP-C1-BtpB (Figure S1) and transfected it into RAW264.7 cells for ectopic expression of BtpB. In agreement with the flow cytometry assay results, more TUNEL-positive cells were observed in the BtpB groups than in the pEGFP-C1 groups at 48 h after being transfected (Figure 1C,D). We also measured cleaved caspase-3, a critical marker of apoptosis. After 48 h, cleaved caspase-3 expression was enhanced significantly in BtpB transfected groups compared with the pEGFP-C1 groups (Figure 1E,F). The results indicate *Brucella* BtpB induced apoptosis in RAW264.7 cells.

### 2.2. Mutant Strain ΔbtpB Infection Increases Autophagic Marker, LC3-II

**Figure 2:**
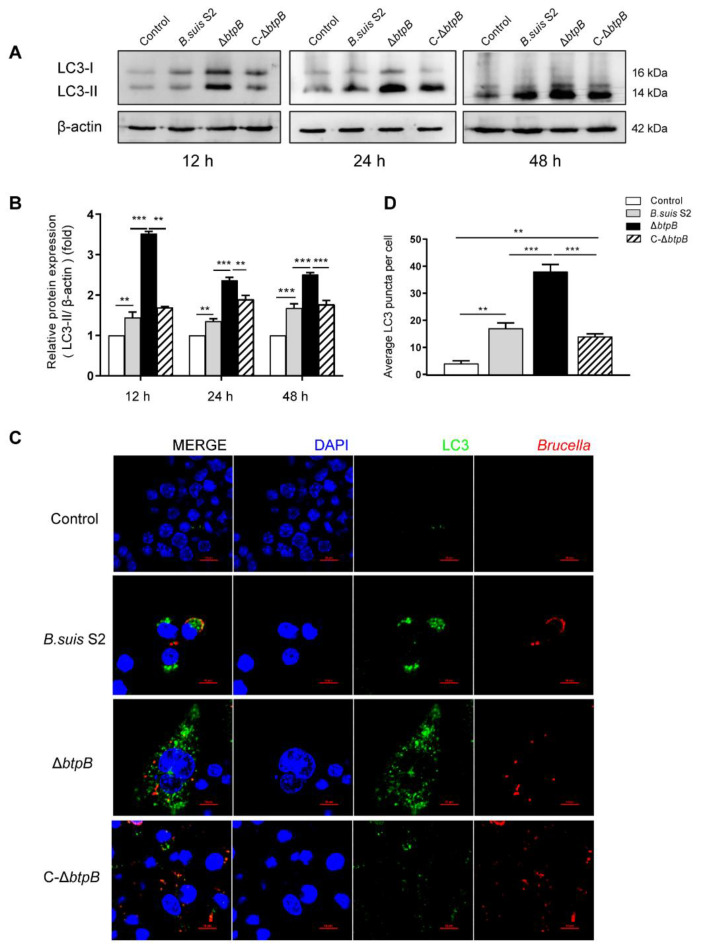
Δ*btpB* promotes the expression of LC3-ΙΙ protein and LC3 puncta. (**A**) RAW264.7 cells were infected with *Brucella* strains (MOI = 200) for 12, 24, and 48 h. At the indicated time point, the LC3 and β-actin expression levels were detected by immunoblotting analysis with specific antibodies. (**B**) The ratio of LC3-ΙΙ relative to β-actin levels was calculated using ImageJ. (**C**) LC3 puncta were detected by confocal immunofluorescence microscopy after *Brucella* infection at 24 h. Scale bars, 10 μm. (**D**) Average number of LC3 puncta per cell in RAW264.7. The experiment was repeated three times. Data represent mean ± SD. ** *p* < 0.01; *** *p* < 0.001, as analyzed with one-way ANOVA and Bonferroni multiple comparison test.

To understand more about other host pathways subject to BtpB subversion, we analyzed the autophagy system. First, we used Western immunoblotting to determine LC3-II protein content, the most commonly used autophagy marker. During autophagy activation, LC3-I is converted to LC3-II, which binds to autophagosome membranes, appearing as LC3 puncta [27]. LC3-II, the lipidated form of LC3, has a larger mass but shows faster mobility in SDS-PAGE, which can be distinguished according to the position of LC3-II (14–16 kDa) and LC3-I (16–18 kDa) by Western blot. As shown in Figure 2A,B, the amount of LC3B-II was upregulated significantly in the group infected with Δ*btpB* compared to *B. suis* S2 and C-Δ*btpB* at 12, 24, and 48 h after infection. This result indicates that lack of *btpB* allows cellular autophagy to proceed. We also detected LC3 puncta by immunofluorescence analysis to further support this conclusion. As expected, infection with the Δ*btpB* mutant led to markedly enhanced LC3 puncta distributed throughout the cytoplasm, whereas the WT and complemented strain infected group showed less LC3 puncta aggregation (Figure 2C,D).

### 2.3. Mutant Strain ΔbtpB Infection Decreases Autophagic Marker, P62

**Figure 3:**
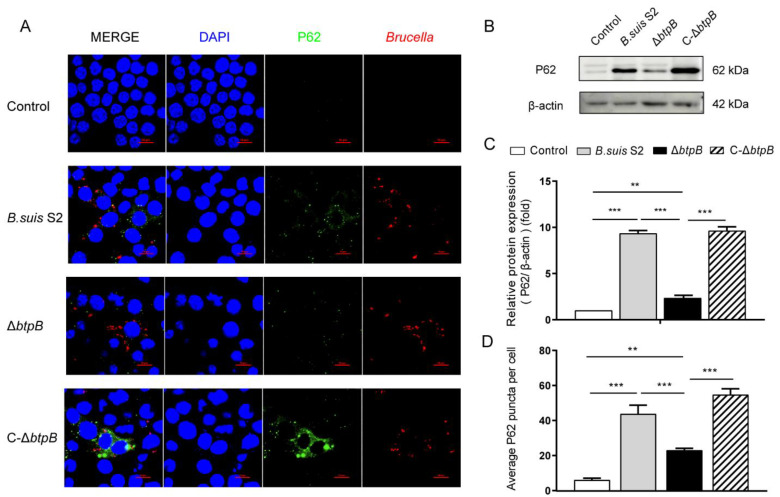
Δ*btpB* inhibits the expression of P62 protein and P62 puncta. RAW264.7 cells were infected with *Brucella* strains (MOI = 200) for 24 h. (**A**) The P62 puncta were imaged by confocal immunofluorescence microscopy at 24 h after *Brucella* infection. Scale bars, 10 μm. (**B**) P62 and β-actin (loading control) expression levels were determined by immunoblotting with specific antibodies. (**C**) ImageJ was used to determine the ratio of P62 relative to β-actin levels. (**D**) The average number of P62 puncta per cell in RAW264.7. The experiment was repeated three times. Data represent mean ± SD. ** *p* < 0.01; *** *p* < 0.001, as analyzed with one-way ANOVA and Bonferroni multiple comparison test.

Along with LC3, P62 is another marker used to reflect the state of autophagy. P62 is also called SQSTM1, which links ubiquitinated proteins to LC3. P62-tagged cargo ultimately routed to the lysosomes to be degraded. Therefore, P62 has a negative correlation with autophagy in most settings. High P62 levels are associated with autophagy inhibition. To further investigate the relationship between BtpB and autophagy, we measured the protein levels of P62. As shown in Figure 3B,C, the Δ*btpB* mutant induced much less P62 protein in RAW264.7 cells compared to *B. suis* S2 and C-Δ*btpB*. We also detected P62 puncta by immunofluorescence; the number of P62 puncta was significantly reduced in the Δ*btpB* group but was more extensive and robust with *B. suis* S2 and C-Δ*btpB*, which is consistent with the immunoblotting results, supporting the hypothesis that Δ*btpB* promotes autophagy (Figure 3A,D).

### 2.4. Mutant Strain ΔbtpB Induces the Accumulation of Autophagolysosomes in RAW264.7

**Figure 4:**
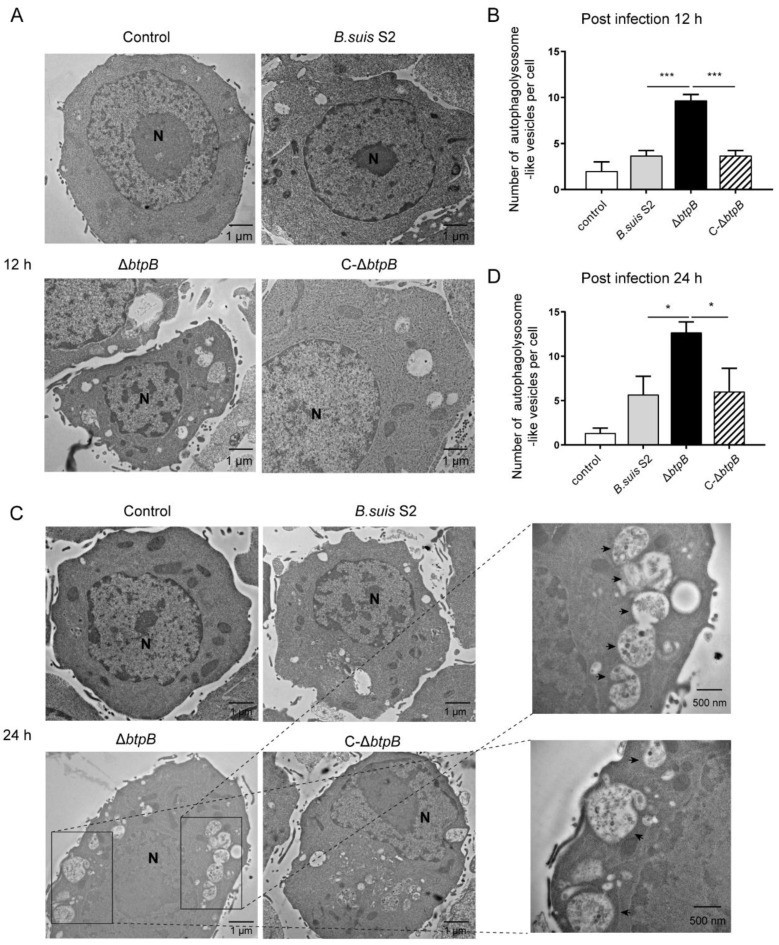
BtpB inhibits autophagolysosome formation during *Brucella* infection. (**A**,**C**) RAW264.7 cells were infected with *Brucella* strains (MOI = 200) for 12 and 24 and samples were imaged by transmission electron microscopy. N, nucleus; black arrowheads indicate autophagolysosomes. Scale bars, 1 μm. (**B**,**D**) Quantification was made according to the average number of autophagolysosomes per cell. Data represent mean ± SD, as analyzed by one-way ANOVA and Bonferroni multiple comparison test. * *p* < 0.05; *** *p* < 0.001.

An accumulation of LC3-positive structures fuses with lysosome to form autophagolysosomes, which then degrade their cargos. To determine whether BtpB affects the downstream autophagy process, we observed the autophagolysosomes in infected cells by transmission electron microscopy. As shown in Figure 4, autophagolysosomes with single-membrane vesicles are widely distributed in the cytoplasm of the Δ*btpB*-infected group, and mostly present a vacuolated structure containing the degraded contents. In comparison to *B. suis* S2 and C-Δ*btpB* groups, the number of autophagolysosomes in the Δ*btpB*-infected group was significantly upregulated at 12 and 24 hpi.

### 2.5. BtpB Blocks Autophagic Flux during Brucella Infection

**Figure 5:**
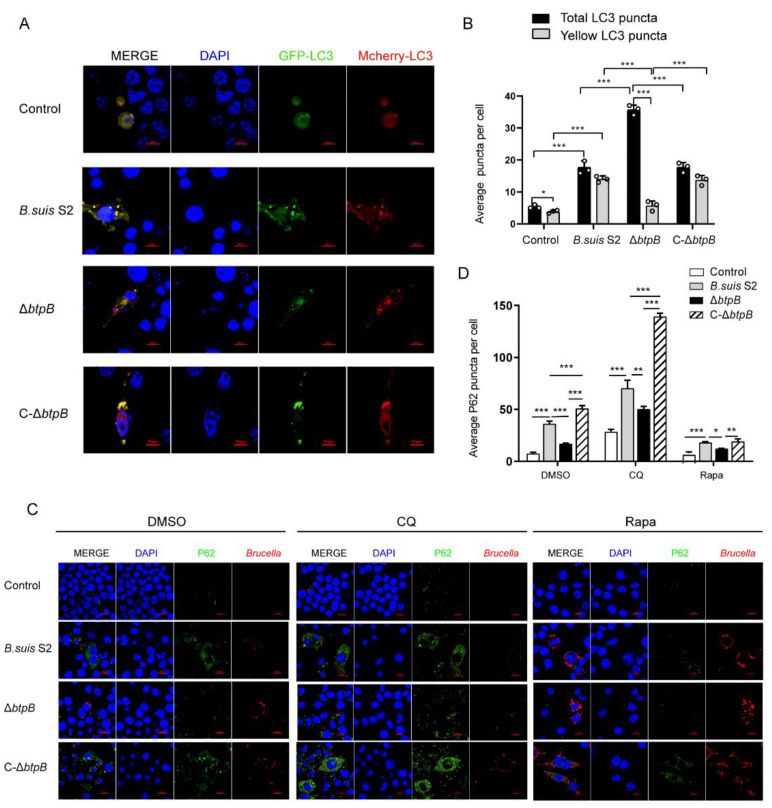
BtpB blocks autophagy flux during *Brucella* infection. (**A**) Representative confocal images of LC3-GFP and LC3-mcherry puncta. RAW264.7 transfected with mCherry-GFP-LC3 for 24 h, then infected with *B. suis* S2, Δ*btpB*, or C-Δ*btpB* (MOI = 200) for 12 and 24 h. Scale bars, 10 μm. (**B**) The average number of LC3 puncta per cell in RAW264.7. (**C**) RAW264.7 cells were infected with *Brucella* strains (MOI = 200) for 24 h and treated with or without 10 μM chloroquine or 10 nM rapamycin. P62 puncta were detected by confocal immunofluorescence microscopy. Scale bars, 10 μm. (**D**) The average number of P62 puncta per cell in RAW264.7. The experiment was repeated three times, and data represent mean ± SD. Two-tailed Student’s *t*-test was used for comparison between two groups. One-way ANOVA and post-hoc Bonferroni multiple comparison test were used to compare more than two groups. * *p* < 0.05; ** *p* < 0.01; *** *p* < 0.001.

An increase in LC3-II and accumulation of autophagolysosomes can be caused by blocking the autophagic flux. The autophagic flux is a dynamic process, involving the formation and degradation of autophagosomes, and the release and reuse of the contents [28]. Thus, we used a tandem reporter GFP-mCherry-LC3 to explore whether Δ*btpB* removes the block to autophagic flux (Figure S2). The GFP fluorescence is sensitive to the acidic lysosomal environment, whereas mCherry is more stable. Hence, yellow puncta indicate immature autophagosomes that have not fused with a lysosome, and red puncta indicate that the autophagolysosomes have activated autophagic flux. The results revealed that Δ*btpB* significantly increased the number of total LC3 puncta relative to *B. suis* S2 and C-Δ*btpB* in RAW264.7 cells, which further confirmed the idea that BtpB inhibits autophagy induction. Moreover, the yellow LC3 puncta were markedly increased in *B. suis* S2- or C-Δ*btpB*-infected macrophages compared to cells infected with the Δ*btpB* strain (Figure 5A,B), confirming that the Δ*btpB* strain can facilitate autophagic flux. The appearance of P62 puncta in the presence of the autophagy inhibitor, chloroquine (CQ), or the autophagy activator, rapamycin (Rapa), was also used to determine the autophagic flux [27]. We found that the number of P62 puncta was remarkably reduced in Δ*btpB* groups but was abundant in *B. suis* S2 and C-Δ*btpB*. Treatment with the inducer of autophagy, Rapa, induced significantly fewer P62 puncta, while the autophagy inhibitor, CQ, resulted in more P62 puncta in the Δ*btpB* group than in *B. suis* S2 and C-Δ*btpB*. These results show that BtpB blocks autophagic flux during *Brucella* infection (Figure 5C,D).

### 2.6. Autophagy Inhibition Decreases Brucella Replication

**Figure 6:**
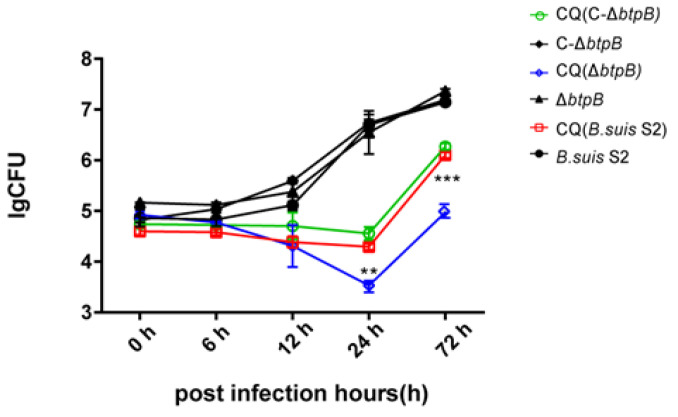
Inhibition of autophagy reduces *Brucella* replication. The growth of *Brucella* strains in RAW264.7 cells treated with 10 μM chloroquine or vehicle. ** *p* < 0.01, *** *p* < 0.001 by one-way ANOVA and post-hoc Bonferroni multiple comparison test.

To analyze the role of autophagy in *Brucella* replication and the effect of BtpB on the intracellular survival of *B. suis* S2, we examined the intracellular viability of *Brucella* strains following chloroquine treatment. As shown in Figure 6, chloroquine significantly decreased *Brucella* CFU counts in RAW264.7 cells after 24 and 72 h. Chloroquine also reduced the intracellular survival of the Δ*btpB* mutant after 12 h and significantly decreased CFU number at 24 hpi, compared to *B. suis* S2 and C-Δ*btpB*.

## 3. Discussion

As a successful intracellular pathogen, *Brucella* has evolved a variety of strategies to subvert host defenses, such as evasion of the host’s antibacterial immune response and phagosome fusion, eventually surviving in the phagosome and causing chronic infection [2,7,29]. Secretion systems encoded by intracellular parasites are key virulence factors that deliver an array of effectors to manipulate the innate immune response, phagosome maturation, apoptosis, vesicular traffic, and autophagy [30,31,32]. At present, 15 effectors of *Brucella* T4SS have been identified. As a T4SS effector, BtpB has a TIR-containing domain that acts to suppress innate immunity [11,12,13]. In this study, we revealed how BtpB manipulated apoptosis and autophagy, which provides new insights into the mechanism of *Brucella* intracellular infection.

Regulation of host-cell apoptosis is an essential step for *Brucella* to achieve its intracellular lifecycle [33]. Inhibition of infected mononuclear cell apoptosis is beneficial to intracellular *Brucella* replication [34,35]. Here, we showed that transfection of RAW264.7 with the *Brucella* BtpB plasmid resulted in significant DNA fragmentation, a typical feature of apoptosis, and upregulation of cleaved caspase-3 expression. Furthermore, flow cytometry showed that Δ*btpB* inhibited macrophage apoptosis compared to *B. suis* S2 and C-Δ*btpB* after 48 h infection. The results prove that BtpB promotes RAW264.7 cell apoptosis, however, the cellular targets and specific mechanisms remain unclear.

Autophagy is a conserved catabolic process for degrading damaged organelles or protein aggregates [36,37]. In addition to its function in maintaining homeostasis, autophagy is also an effective way of eliminating intracellular pathogens by lysosomal digestion; however, microbes have evolved strategies to modulate or hijack autophagy for their survival [32,38,39]. For example, SseG and SseF, the *Salmonella* SPI2-T3SS effectors, inhibited autophagy and promoted intracellular pathogen replication via their direct interaction and inactivation of Rab1A [40]. *Mycobacterium tuberculosis* PknG promoted autophagy but inhibited autophagosome maturation, resulting in blockage of autophagic flux and enhanced intracellular survival of pathogens [41]. It is known that the *Brucella* intracellular cycle selectively co-opts autophagy-associated proteins to subvert host clearance and promote infection [8,42]; however, the mechanism of how bacterial virulence factors regulate autophagy is not fully understood. 

In this study, we showed that the *Brucella* effector BtpB inhibited autophagy. The protein level of the traditional autophagy marker, LC3-II, was markedly enhanced by Δ*btpB* compared to the parental strain *B. suis* S2 and the complemented strain C-Δ*btpB*. Detection of LC3 puncta by immunofluorescence also supported that conclusion. In the autophagy process, LC3-decorated autophagosomes bind to SQSTM1/P62 and undergo fusion with lysosomes resulting in a compartment termed the autophagolysosome, in which the cargos are degraded. The results of our TEM experiment revealed that the number of autophagolysosomes was markedly increased in the cells infected with the Δ*btpB* strain, which meant the autophagolysosome generated more or degraded less. To better understand this result, we determined the level of autophagic flux by means of the specially designed mCherry-GFP-LC3B fusion fluorescent protein. We found that Δ*btpB* reduced the yellow LC3 puncta and promoted autophagic flux. We also reached the same conclusion in the LC3-II turnover assay, which measured the difference in LC3-II amounts in the presence of an inhibitor that neutralized the lysosomal pH compared to vehicle. An increase in LC3-II in the presence of the inhibitor indicated that flux had occurred [17]. We found that Δ*btpB* induced more LC3 and less P62 than *B. suis* S2 and C-Δ*btpB* in RAW264.7 cells treated with CQ, supporting the hypothesis that the Δ*btpB* strain facilitated autophagic flux. According to the guidelines for monitoring autophagy, immunostaining of P62 with autophagy inducers and inhibitors is another valuable tool for monitoring autophagy flux [17]. Our data showed that the inhibitor CQ induced more P62 puncta, while the inducer, Rapa, led to fewer P62 puncta in Δ*btpB*-infected cells than in *B. suis* S2 and C-Δ*btpB* groups. These experiments proved that Δ*btpB* promoted autophagy and the formation of autophagolysosomes, further leading to an overall effect of activated autophagic flux.

Manipulation of autophagic flux is a pivotal way for intracellular pathogens to ensure their survival [43]. To further determine whether BtpB manipulated intracellular proliferation of *Brucella* under altered autophagy conditions, we measured the growth of the *Brucella* strains with chloroquine treatment, which has been shown to inhibit autophagy and lysosomal fusion. The data revealed that CQ inhibited intracellular growth of *Brucella*, and the mutant strain Δ*btpB* showed a lower CFU than *B. suis* S2 and C-Δ*btpB*. The immunostaining of *Brucella* in Figure 4 also supported that conclusion. The results indicate that BtpB promotes intracellular proliferation of *Brucella* under conditions of autophagy inhibition. We speculate that activation of autophagy flux is beneficial to *Brucella* survival. Therefore, BtpB might be needed for *Brucella* intracellular survival when autophagy is inhibited; however, this conclusion remains to be proven by additional experiments.

In conclusion, our results revealed that the T4SS effector BtpB was harmful, causing DNA breakage and triggering apoptosis of RAW264.7 cells. Moreover, we demonstrated that BtpB possesses the ability to inhibit autophagy, block the progression of autophagic flux, and decrease autophagolysosome formation during *Brucella* infection. Simultaneously, BtpB can manipulate *Brucella* intracellular survival in an environment where autophagy is inhibited. These findings constitute a new mechanism for how BtpB subverts the host’s defenses against *Brucella* and provide new insights for potential *Brucella* treatment strategies.

## 4. Materials and Methods

### 4.1. Bacterial Strains and Mammalian Cells

The *B. suis* S2 (CVCC70502) vaccine was provided by the Veterinary Drug Control Institute of Shaanxi Provincial (Xi’an, Shaanxi, China). The *B. suis* S2 derivative strains were constructed in our laboratory [12]. All *Brucella* strains were grown on tryptic soy broth agar (TSA) for 72 h or in tryptic soy broth (TSB) with shaking for 48 h. The RAW264.7 cells were cultured in DMEM with 10% fetal bovine serum (ThermoScientifc, Waltham, MA, USA) at 37 °C with 5% CO_2_.

### 4.2. DNA Constructs

The *btpB* gene sequence was retrieved from the Genbank database. *B. suis* S2 genomic DNA was used as a template for PCR amplification of the *btpB* gene. After gel recovery, the fragment was digested with *BamH* I and ligated into pEGFP-C1 to construct pEGFP-C1-BtpB.

For autophagy flux analysis, the plasmid pEGFP-mCherry-LC3B was constructed. PCR was used to amplify the LC3B gene from mouse cDNA with the LC3B F/R primers, and the mCherry gene was amplified from pmCherry-C1 with mCherry F/R primers (Table 1). Subsequently, the two amplicons were ligated by overlap-PCR. The purified product was digested with *BamH* I and then inserted into pEGFP-C1 to construct the recombinant plasmid, pEGFP-mCherry-LC3B.

### 4.3. Transfection

RAW264.7 macrophages were seeded into 24-well plates at 2 × 10^5^ cells per well. The cells were routinely transfected with the plasmids using the X-tremeGENE HP DNA transfection reagent (Roche, Basel, Switzerland). Briefly, cells at 70% confluency, plasmid, and transfection reagent were added into Opti-MEM (TermoScientifc, Waltham, MA, USA) and incubated 20 min at RT, then transferred to cell culture incubator at 37 °C.

### 4.4. Macrophage Infection Assay

The macrophage infection assays were done as described previously [12]. Briefly, RAW264.7 cells were seeded into 6-well plates at 1 × 10^6^ cells per well and cultured for 12 h. Cultures of three strains of bacteria were centrifuged at 5500× *g* for 15 min and washed with sterile PBS, then resuspended and plated on TSA to count colony-forming units (CFU). The cells were inoculated with *B. suis* S2, Δ*btpB*, or C-Δ*btpB* at a multiplicity of infection (MOI) of 200. After 4 h, the cells were washed three times with PBS and cultured in DMEM containing 50 μg/mL of gentamicin for 1 h to eliminate extracellular bacteria. Then, the medium was replaced with DMEM containing 10% FBS and 25 μg/mL gentamicin. At this point, chloroquine (10 mΜ, MCE) or rapamycin (10 nM, MCE) were added where required.

### 4.5. Transmission Electron Microscopy (TEM)

RAW264.7 cells were cultured to logarithmic phase (60–80% confluence) and inoculated with *B. suis* S2, Δ*btpB*, or C-Δ*btpB* at an MOI of 200 for 12 and 24 h. The cells were washed with PBS and centrifuged at 800× *g* for 5 min, then pre-fixed with 2.5% glutaraldehyde in phosphate buffer (0.1 M, pH 7.2–7.4) at 4 °C overnight. After washing with PBS, cells were incubated for two hours with 1% O_S_O_4_ in PB. Fixed cells were dehydrated with ethanol at concentrations of 30, 50, 70, 80, 90, and 100 percent for 8 min each and infiltrated in resin. The resin was polymerized at 55 °C for two days, then sectioned (50–70 nm) and stained for 20 min with 2% uranyl acetate and 10 min with lead citrate and observed by TEM (Technai G2, Spirit Bio, FEI, Hillsboro, OR, USA).

### 4.6. CFU Counting of Infected RAW264.7 Macrophages

RAW264.7 cells were inoculated with *B. suis* S2, Δ*btpB*, or C-Δ*btpB* at an MOI of 200. At the indicated points, cells were washed three times with PBS and lysed with PBS containing 0.5% Triton X-100 for 10 min. Lysates were serially diluted with PBS, plated on TSA, and incubated at 37 °C for 72 h.

### 4.7. TUNEL Apoptosis Assay

DNA fragmentation was detected using a terminal deoxynucleotidyl transferase-mediated dUTP nick end labeling (TUNEL) kit (Beyotime, C1090, Shanghai, China). After washing with PBS, the infected cells were fixed with 4% paraformaldehyde (PFA) and permeabilized with 0.2% Triton X-100 for 5 min. After washing twice with PBS, the cells were incubated with TUNEL reagent for 1 h at 37 °C and imaged under a fluorescence microscope (Ni-U, Nikon, Tokyo, Japan) at 488 nm excitation/530 nm emission.

### 4.8. Flow Cytometry Analysis

The percentage of apoptotic cells was determined by flow cytometry (Keygen, KGA105, Nanjing, China). In brief, after infection with *Brucella* strains, cells were centrifuged, washed three times with PBS, and incubated with 5 μL of FITC-labeled Annexin V and 5 μL of PI for 15 min at room temperature in darkness. The cells were analyzed by flow cytometry (BD FACSAria™ III, Franklin Lakes, NJ, USA) and data were evaluated with Flowjo. For each determination, a minimum of 30,000 cells were analyzed.

### 4.9. Western Blotting

Infected macrophages were centrifuged, washed with PBS, and disrupted in lysis buffer (Keygen, KGP10100, Nanjing, China) in an ice-bath for 30 min. The lysates were centrifuged at 12,000× *g*, 4 °C for 15 min and supernatants were collected. Protein concentrations were determined by a BCA kit (Keygen, KGP903, Nanjing, China). SDS loading buffer was added, and samples were boiled at 100 °C for 5 min. Protein samples were separated by 12% SDS-PAGE and blotted onto PVDF membranes. The membranes were blocked with 10% nonfat powdered milk for 2 h and exposed to the relevant primary antibody for 24 h at 4 °C: rabbit anti-cleaved caspase-3 (1:1000; catalog number 9661; CST, Danvers, MA, USA), rabbit anti-LC3B (1:1000; catalog number ab192890; Abcam, Cambridge, UK), rabbit anti-P62 (1:1000; catalog number ab109012; Abcam), mouse anti-β-actin (1:5000; catalog number ab8226; Abcam). After washing, the blots were incubated with HRP-conjugated secondary antibody at RT for 2 h. The signal was visualized using an ECL chemiluminescence kit (Beyotime, P0018FS, Shanghai, China), and imaged by a gel imaging system (GE Amersham Imager 800, Boston, NY, USA).

### 4.10. Immunofluorescence

For immunofluorescence analyses, the macrophages were seeded in 24-well plates on glass coverslips. After 24 h incubation with *Brucella* strains, the cells were fixed with 4% paraformaldehyde and permeabilized with 0.2% Triton X-100 in PBS for 20 min at room temperature, and then washed three times with PBS. The slides were incubated with anti-LC3B (1:500, ab192890, Abcam), anti-P62 (1:500, ab109012, Abcam), or anti-*Brucella* (1:500) at 4 °C overnight and then stained with Alexa-Fluor 488-conjugated donkey anti-rabbit IgG (for LC3B and P62, 1:1000, A32790, ThermoFisher) and Alexa-Fluor 555-conjugated donkey anti-goat IgG (for *Brucella*, 1:1000, A-21432, ThermoFisher) for 1 h, at RT. Nuclei were stained with DAPI for 10 min. The slides were washed four times with PBS after each incubation. Samples were captured by a confocal microscope (A1R; Nikon, Tokyo, Japan).

### 4.11. GFP-mCherry-LC3 Assay

RAW264.7 macrophages were transfected with the plasmid pEGFP-mCherry-LC3B using X-tremeGENE HP DNA transfection reagent for 24 h. After infection with *Brucella* strains following the procedure, the cells were fixed with 4% PFA and imaged with a confocal microscope (A1R; Nikon, Tokyo, Japan).

### 4.12. Statistical Analysis

Data analyses were performed using SPSS. Two-tailed Student’s *t*-tests were used for group comparisons. One-way ANOVA with Bonferroni multiple comparison test was performed for significance assessment among three or more groups. Statistical significance was established as *p* < 0.05.

## Figures and Tables

**Table 1:** Primers used in this study.

Primers	Sequences (5′-3′)
BtpB F	GGTACCGCGGGCCCGGGATCCATGTACAATTTATTTGTTTCG (*BamH* I)
BtpB R	TTATCTAGATCCGGTGGATCCCTAGGTGATGAGGGCGACGCGCTC (*BamH* I)
mCherry F	GTACCGCGGGCCCGGGATCCATGGTGAGCAAGGGC (*BamH* I)
mCherry R	cttctcggacggcatCTTGTACAGCTCGTC
LC3B F	GACGAGCTGTACAAGatgccgtccgagaag
LC3B R	TATCTAGATCCGGTGGATCCttacacagccattgct (*BamH* I)

## Data Availability

Not applicable.

## Supplementary Materials

The following supporting information can be downloaded at: https://www.mdpi.com/article/10.3390/ijms232214439/s1.
