# Supplementary material for: Brucella BtpB Manipulates Apoptosis and Autophagic Flux in RAW264.7 Cells
Source: Int J Mol Sci. 2022 Nov 20;23(22):14439. doi: 10.3390/ijms232214439 (PMC9693124; doi:10.3390/ijms232214439)
Supplement: Supplementary file 1 [file ijms-23-14439-s001.zip › ijms-1939012-supplementary.pdf]

Supplemental information

Supplemental Figures 1-2

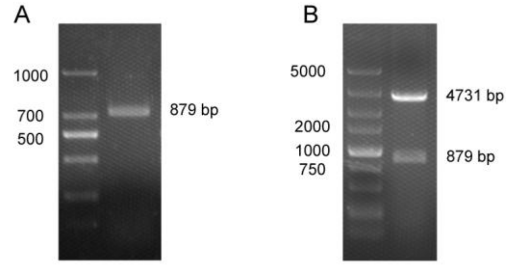

Figure S1: A. *btpB* gene from *B. suis* S2 genomic DNA was amplified by PCR. B. Confirmation of the pEGFP-C1-BtpB recombinant plasmid by digestion with the restriction enzyme *Bam*H I.

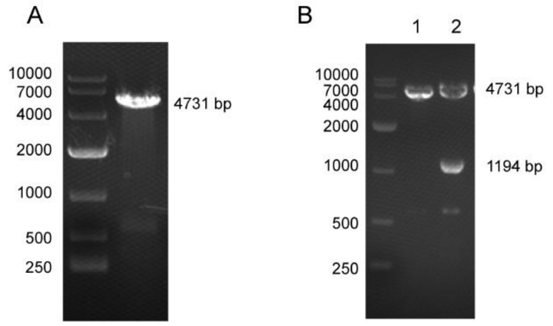

Figure S2: A. Linearization of the pEGFP-C1 plasmid by digested with the restriction enzyme *Bam*H I. B. The pEGFP-mCherry-LC3B recombinant plasmid is identified by digestion with the restriction enzyme *Bam*H I. Lane 1, the PEGFP-C1 plasmid; Lane 2, mCherry + LC3.
